# Effects of Low-Carbohydrate Diet and Exercise Training on Gut Microbiota

**DOI:** 10.3389/fnut.2022.884550

**Published:** 2022-05-03

**Authors:** Shengyan Sun, On Kei Lei, Jinlei Nie, Qingde Shi, Yuming Xu, Zhaowei Kong

**Affiliations:** ^1^Institute of Physical Education, Huzhou University, Huzhou, China; ^2^Faculty of Education, University of Macau, Macao, Macao SAR, China; ^3^Faculty of Health Sciences and Sports, Macao Polytechnic University, Macao, Macao SAR, China; ^4^College of Physical Education, Hangzhou Normal University, Hangzhou, China

**Keywords:** ketogenic diet, high-intensity interval training, moderate-intensity continuous training, microbiome, obesity

## Abstract

**Objective:**

This study was aimed to evaluate the effects of low-carbohydrate diet (LC) and incorporated high-intensity interval training (HIIT) or moderate-intensity continuous training (MICT) on gut microbiota, and the associations between changes in gut microbiota and cardiometabolic health-related profiles.

**Methods:**

Fifty overweight/obese Chinese females (age 22.2 ± 3.3 years, body mass index 25.1 ± 3.1 kg/m^–2^) were randomized to the groups of LC, LC and HIIT (LC-HIIT, 10 repetitions of 6-s sprints and 9-s rest), and LC and MICT group (LC-MICT, cycling at 50–60% V̇O_2peak_ for 30 min). The LC-HIIT and LC-MICT experienced 20 training sessions over 4 weeks.

**Results:**

The 4-week LC intervention with/without additional training failed to change the Shannon, Chao 1, and Simpson indexes (*p* > 0.05), LC increased *Phascolarctobacterium* genus, and LC-HIIT reduced *Bifidobacterium* genus after intervention (*p* < 0.05). Groups with extra exercise training increased short-chain fatty acid-producing *Blautia* genus (*p* < 0.05) and reduced type 2 diabetes-related genus *Alistipes* (*p* < 0.05) compared to LC. *Sutterella* (*r* = −0.335) and *Enterobacter* (*r* = 0.334) were associated with changes in body composition (*p* < 0.05). Changes in *Ruminococcus*, *Eubacterium*, and *Roseburia* genera were positively associated with blood pressure (BP) changes (*r* = 0.392–0.445, *p* < 0.05), whereas the changes in *Bacteroides*, *Faecalibacterium*, and *Parabacteroides* genera were negatively associated with BP changes (*r* = −0.567 to −0.362, *p* < 0.05).

**Conclusion:**

LC intervention did not change the α-diversity and overall structure of gut microbiota. Combining LC with exercise training may have additional benefits on gut physiology. Specific microbial genera were associated with LC- and exercise-induced regulation of cardiometabolic health.

## Introduction

Given that genetic and environmental (lifestyle) factors do not fully explain the prevalence of obesity, recent efforts to combat obesity and related metabolic disorders have focused on the role of gut microbiota in obesity incidence and the identification of key microbial targets associated with obesity and associated metabolic disorders (1–5). A state of bacterial dysbiosis was observed both in obese mice and humans (1, 4), which is associated with a greater capacity for energy harvest, thereby contributing to obesity (1). In support, germ-free mice were found to be more resistant to diet-induced obesity, insulin resistance, and dyslipidemia than their conventional siblings when exposed to a high-fat diet (2, 3). Moreover, after transplantation of the microbiota from conventional mice, the amount of body fat in the originally germ-free mice increased by 66% within 2 weeks despite a reduced energy intake, accompanied by decreased insulin sensitivity and adipocyte hypertrophy (2). These findings indicate a causal effect of gut microbiota in host energy storage, metabolic homeostasis, and the incidence of obesity and associated metabolic disorders.

As a popular diet approach with drastic reduction in carbohydrate intake, low-carbohydrate diet (LC) was frequently used to control weight and improve cardiometabolic health (6–9). Given that high levels of dietary fat and protein fermentation by gut microbiota have been associated with higher fecal endotoxin levels (10, 11), some studies reported adverse effects of LC on gut microbiota, including diminished total bacterial levels (12), lowered α-diversity (13), enriched proinflammatory bacterial species (14), and decreased anti-inflammatory microbial species (15). Simultaneously, LC increased presumptively beneficial gut microbiota, decreased pro-inflammatory genera (16), and corrected imbalanced gut microbiota (13, 17) was also reported. Given these inconsistent findings, more studies are needed to evaluate the effects of LC on gut microbiota and the role of gut microbiota in LC-induced cardiometabolic health changes.

In contrast, findings regarding the effect of exercise training on gut microbiota are more consistent. Studies both in rodents (18, 19) and humans (20, 21) showed that the traditional endurance-based exercise training or called moderate-intensity continuous training (MICT) could increase gut microbial diversity, enrich beneficial microbial members and fecal metabolites, and modify gut physiology to a favorable way. And these favorable gut microbial changes were closely associated with exercise prevention of high fat diet-induced obesity (18). High-intensity interval training (HIIT) is considered as a time-efficient alternative to MICT in improving cardiometabolic health; however, the effect of HIIT on gut microbial modification in human obesity are currently unknown and warrant further study.

In our previous studies, LC intervention reduced body mass and blood pressure (BP), and improved body composition and insulin sensitivity; when LC was combined with extra HIIT or MICT, additional improvement in cardiorespiratory fitness (CRF) was gained (6–8). Based on these findings, it is interesting to further examine whether the addition of HIIT and MICT would also induce additional benefits on gut microbiota during LC intervention. As a result, the primary objective of this randomized controlled trial was to verify the effect of LC on gut microbiota in overweight/obese women, and to examine whether combined LC with exercise training (i.e., HIIT and MICT) could reverse the potential adverse effect of LC on gut microbiota, or could trigger additional benefits. The secondary objective was to examine whether changes in cardiometabolic risk factors would be associated with concurrent changes in gut microbiota, and to recognize the potential microbial targets.

## Methods

### Participants and Experimental Design

The study protocol was conducted according to the Declaration of Helsinki and approved by the Research Ethics Committee of the University of Macau (RC Ref. no. MYRG2017-00199-FED). Before recruiting, sample size calculation was performed using G*Power (Version 3.1), under the assumptions of a correlation of 0.8 between pre–post intervention measurements and an effect size of 0.32 based on a meta-analysis for the primary outcome of peak oxygen uptake (V̇O_2peak_) resulting from HIIT (22). The results showed that 12 participants per group were required. Recruitment notices with research introductions and inclusion criteria were posted on bulletin boards on campus and in dormitories to recruit overweight/obese but healthy women. The 50 enrolled female participants were between 18 and 30 years old, healthy (no endocrine, metabolic, osteoarticular, or cardiovascular diseases) but initially overweight or obese with a body mass index (BMI) over 23 kg/m^–2^ (23). They were not alcohol users or smokers, not participating in organized training programs or specific eating plans at recruitment, not taking any prescribed drugs, antibiotics treatment, and weight loss and nutritional supplements in the past 3 months, and having a stable weight (variation within 5%) in the past 6 months. All participants provided written informed consents and were randomly assigned to either an LC group (LC, *n* = 16), an LC and HIIT group (LC-HIIT, *n* = 17), or an LC and MICT group (LC-MICT, *n* = 17). Excluding the dropouts, 11, 13, and 12 participants, respectively, in LC, LC-HIIT, and LC-MICT groups who completed the whole trial were finally included in data analysis.

The LC group took 2 weeks of normal diet as baseline, and then switched to LC for 4 weeks after pre-tests, followed by post-intervention tests. The LC-MICT and LC-HIIT groups were on the same diet plan, but additionally received supervised MICT or HIIT 5 days⋅week^−1^ in the meanwhile.

### Dietary Protocol

During the 2-week normal diet period, participants maintained their normal diet and kept 3-day food diaries (2 weekdays and 1 weekend day) to calculate their baseline daily energy intakes and macronutrient compositions using the nutrition analysis and management system (NRISM, version 3.1, Beijing, China). During the 4-week LC period, participants were instructed to retain their daily energy intake but switch to LC. Detailed instructions on how to record food diaries and how to perform LC were given to each participant individually by a dietitian. An LC operation manual listing appropriate food/drink for LC recipes as well as points for attention was also provided (8).

### Exercise Training Protocol

Before each training session, 2–3 min of free stretching was performed as warm-up. The LC-HIIT group performed repeated sprint cycling exercise on cycle ergometers (Monark 894E, Varberg, Sweden). Participants cycled as fast as possible for 6 s against an initial resistance of 1 kg, and rested on seat for 9 s as recovery, the sprinting and rest bouts were repeated 10 times in one training session (2.5 min⋅session^−1^) (8). The LC-MICT group performed 30-min continuous cycling exercise on an ergocycle (Ergometer 900PC, Ergoline, Germany) at a pedaling speed of 50 ± 5 rpm. The exercise intensity was 50% of V̇O_2peak_ of pre-test for the first 2 weeks and increased to 60% of pre-V̇O_2peak_ for the last 2 weeks.

### Pre- and Post-intervention Assessments

The pre- and post-intervention measures of anthropometric indexes, BP, CRF, and gut microbiota were carried out 48–120 h before the first intervention day and 72–96 h after the last intervention day, respectively.

### Anthropometric Indexes and Blood Pressure

Body mass index (in kg/m^–2^) was calculated using body weight and height measured by a wall-mounted stadiometer and an electronic scale with bare feet and in light clothing. Waist circumference (WC) was measured at the midpoint between the lower edge of the rib cage and the iliac crest, while the maximum circumference over the buttocks was measured as hip circumference (HC). Waist-to-hip ratio (WHR) was calculated as WC divided by HC. BP was measured two times using an electronic BP monitor (Microlife 3BTO-A, Taipei, Taiwan) on participant’s left arm in a seating position; the mean value of the two tests was taken as systolic blood pressure (SBP) and diastolic blood pressure (DBP). Mean arterial pressure (MAP) was calculated as (SBP + 2 × DBP)/3.

### Maximal Incremental Exercise Test

After a brief warm-up, participants started to pedal on an electric-braked cycle ergometer (Monark 839E, Sweden) against 50 W with the rate of 60 ± 5 rpm. The workload was increased 25 W every 3 min until volitional exhaustion. Respiratory gases were continuously assessed using a gas analyzer (Vmax Encore System, CareFusion Corp., San Diego, CA, United States). The largest oxygen consumption value averaged over 15 s of the last exercise stage was calculated as V̇O_2peak_ (24).

### Fecal DNA Extraction, Amplification, Sequencing, and Bioinformatics

Dry, clean, and sterilized containers were provided to participants to collect fecal samples. Fecal samples were collected from evacuated stool by the participants according to detailed instructions. Five to 10 grams of fresh feces form different parts were taken using the dedicated scoop provided. The fecal samples were then immediately stored at −80°C for further processing.

Total bacterial DNA from fecal samples was extracted using GTX Stool Extraction Kits (Hain Lifescience, Nehren, Germany) following the manufacturer’s instructions. The size and integrity of extracted DNA were evaluated by 1% agarose gel electrophoresis, and the DNA concentration was quantified using the Qubit 2.0 Fluorometer (Life technologies, Grand Island, NY, United States). Further sequencing of the DNA samples and bioinformatics service were provided by KingMed Diagnostics Co. (Guangzhou, China). The V3–V9 hypervariable regions of the 16S rRNA gene in bacteria were PCR-amplified using 16S Ion Metagenomics Kit (ThermoFisher Scientific, Shanghai, China, Cat. no. A26216). The amplified fragments were purified, quantified, and sequenced on the Ion PGM™ System and analyzed with the Ion Reporter™ software (Ion 16S™ Metagenomics Kit analyses module). Raw sequencing data were processed using the QIIME software package (v.1.9) (25). The sequence readings were quality-screened with a quality score over 20 bp, and then clustered into operational taxonomic units (OTUs) with a 97% similarity threshold against the Greengenes database (26). After obtaining the OUT tables, species annotation and taxonomic analysis of the representative OTU sequences were assigned using RDP classifier (version 2.2). In community ecology studies, α-diversity can reflect the abundance, uniformity, and diversity of microbial communities. In this study, three estimators evaluating the diversity and richness of gut microbial community were calculated using MOTHUR (version 1.5.0), namely, the Shannon (diversity estimator), Chao 1 (species-based richness estimator), and Simpson indexes (diversity estimator). The bacteria with a relative abundance larger than 1% of the total microbiota were considered reliable and used for further analyses.

### Statistical Analysis

Statistical analyses were performed using the PASW software (Release 22.0; IBM, New York, NY, United States). Prior to the main statistical analyses, the Shapiro–Wilk test was performed to confirm the normal distribution of outcome variables. Two-way repeated measures of ANOVA were conducted to determine the main effects (i.e., time and group) and interaction effects (time × group). When significant interaction and main effects were observed, Tukey’s honestly significant difference *post hoc* tests were performed to identify the difference among groups.

Principal coordinate analysis (PCA) based on Bray–Curtis distance was performed to compare the overall gut microbial composition among different groups before and after intervention at the genus level. Two-way permutational multivariate analysis of variance (PERMANOVA) was conducted to examine the differences in gut microbial composition among groups at phylum and genus levels on pre- and post-intervention. For significant interaction effects, or main effects of time and group, *post hoc* tests (one-way PERMANOVA with Bonferroni correction, and Wilcoxon test) were further conducted to examine the differences among groups, and differences in values before and after intervention. The statistical analyses of PCA and PERMANOVA were conducted using the PAST software (version 3.25).

Pearson’s correlation tests were performed in the whole cohort to examine the associations between changes in the relative abundance of microbial genera and changes in cardiometabolic health-related profiles. Correlation coefficient (*r*) ≤ 0.1 was regarded as a weak or small correlation, 0.3 ≤ *r* < 0.5 meant a moderate correlation, and *r* ≥ 0.5 was considered a strong or large correlation (27). Data were presented as means (standard deviations, SDs), and *p* < 0.05 with two tails was set as statistical significance.

## Results

### Changes in Cardiometabolic Health After Low-Carbohydrate and Training Intervention

Overall, the overweight/obese young females were 21.6 ± 3.4 years old with an initial BMI of 24.8 ± 2.4 kg/m^–2^ (height: 162.1 ± 5.1 cm, weight: 65.3 ± 8.1 kg). After intervention, all three groups experienced remarkable decrements in body weight (*p* < 0.01, η^2^ = 0.772), BMI (*p* < 0.01, η^2^ = 0.782), WC (*p* < 0.01, η^2^ = 0.717), and HC (*p* < 0.01, η^2^ = 0.763, Table 1). Specifically, the LC, LC-HIIT, and LC-MICT groups reduced body weight by 2.5 ± 1.8, 2.7 ± 1.3, and 2.4 ± 1.3 kg, respectively. Moreover, similar reductions in SBP (*p* < 0.01, η^2^ = 0.370) and MAP (*p* < 0.01, η^2^ = 0.321) were found in all groups without group differences. In contrast, V̇O_2peak_ was only improved in groups with additional training (LC-HIIT increased 3.4 ± 2.2 ml⋅min^−1^⋅kg^−1^, and LC-MICT increased 3.7 ± 3.0 ml⋅min^−1^⋅kg^−1^). These data were published in our previous study (7, 8).

**TABLE 1:** Main outcome variables before and after LC with/without training (the cardiometabolic outcomes are published data).

	LC (*n* = 11)		LC-HIIT (*n* = 13)		LC-MICT (*n* = 12)	
	Pre	Post	Pre	Post	Pre	Post
Age (years)	21.6 (4.3)		21.4 (2.9)		21.8 (3.1)	
Height (cm)	161.4 (4.1)		163.5 (6.4)		161.1 (4.4)	
Weight (kg)	64.6 (9.3)	62.1(8.3)^∧^	66.7 (8.8)	64.0(8.3)^∧^	64.4 (6.7)	61.9(6.3)^∧^
BMI (kg/m^–2^)	24.8 (3.3)	23.8(3.1)^∧^	24.8 (2.0)	23.9(1.9)^∧^	24.8 (1.9)	23.8(1.9)^∧^
WC (cm)	76.4 (8.5)	73.0(7.0)^∧^	77.8 (6.4)	73.7(6.2)^∧^	75.6 (6.5)	71.4(4.9)^∧^
HC (cm)	100.6 (4.8)	97.3(5.1)^∧^	100.0 (4.6)	98.1(4.9)^∧^	100.7 (5.2)	97.6(5.6)^∧^
WHR	0.76 (0.05)	0.75 (0.04)	0.78 (0.04)	0.75(0.04)^∧^	0.75 (0.04)	0.73(0.03)*
SBP (mmHg)	113 (11)	108(9)*	111 (8)	106(13)*	111 (8)	106(10)*
DBP (mmHg)	71 (9)	69 (8)	70 (6)	66(8)*	71 (5)	68 (9)
MAP (mmHg)	85 (9)	82 (8)	84 (6)	79(9)*	84 (5)	80(9)*
V̇O_2peak_ (ml⋅min^−1^)	1.70 (0.18)	1.68 (0.21)	1.58 (0.26)	1.73(0.25)^†∧^	1.48 (0.29)	1.70(0.28)^†∧^
V̇O_2peak_ (ml⋅min^−1^⋅kg^−1^)	26.6 (5.0)	26.4 (2.6)	23.7 (2.4)	26.9(3.6)^†∧^	23.3 (4.6)	27.2(3.9)^†∧^
Shannon	3.02 (0.68)	3.32 (0.86)	3.19 (0.69)	3.30 (0.86)	3.54 (0.53)	3.71 (0.41)
Simpson	0.76 (0.13)	0.79 (0.19)	0.77 (0.15)	0.78 (0.17)	0.84 (0.09)	0.87 (0.08)
Chao 1	55.59 (11.13)	62.75 (13.49)	54.31 (9.46)	59.01 (16.65)	58.21 (8.95)	67.28 (6.61)

Outcome variables are presented as mean (SD). LC, low-carbohydrate diet group; LC-HIIT, low-carbohydrate diet and high-intensity interval training group; LC-MICT, low-carbohydrate and moderate-intensity continuous training group. Within-subjects comparison from pre- and post-measures at *p < 0.05, ^∧^p < 0.01; comparison with LC at ^†^p < 0.05.

### Dietary Compositions and Daily Physical Activities

At baseline, participants took approximately 2,000 kcal⋅day^−1^, in which ∼45.0, ∼15.0, and ∼40.0% of the energy intake were derived from carbohydrates, proteins, and fats, respectively. During the LC intervention, total energy intake was non-significantly reduced to ∼1,900 kcal per day, with carbohydrates, proteins, and fats accounting for ∼9.0, ∼23.0, and ∼68.0% of daily energy intakes. Protein and fat consumptions were significantly higher (*p* < 0.01), whereas dietary carbohydrate was significantly lower (*p* < 0.01) compared to baseline, but no group difference was observed. There were no differences on daily physical activities among the three groups as measured by pedometers (Yamax Digi-Walker SW-200, Tokyo, Japan) throughout the experiment. Detailed data were published previously (8).

### Effects of Low-Carbohydrate and Training Intervention on Gut Microbiota

**FIGURE 1:**
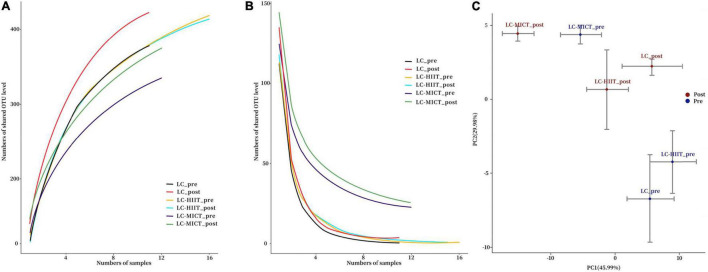
Pan analysis curve **(A)**, Core analysis curve **(B)**, and PCA plot of the microbial populations **(C)** before and after intervention.

The Pan and Core analysis curves are presented in Figures 1A,B. The community diversity indicators (Shannon and Simpson indexes) and richness indicator (Chao 1) showed that the α-diversity of gut microbiota was unaffected by LC and/or training intervention (*p* > 0.05, Table 1). The PCA plot showed that there was no clear group clustering of microbial populations before and after LC intervention with/without exercise training (Figure 1C). In terms of alterations in the gut microbial taxonomy, no phylum-level alterations in the relative abundance of gut microbiota were observed (*p* > 0.05, Table 2). At the genus level, though significant time effects were observed in *Clostridium*, *Bifidobacterium*, *Phascolarctobacterium*, and [*Ruminococcus*] genera, the *post hoc* tests showed that *Phascolarctobacterium* (*p* < 0.05) members were significantly increased in the LC group, whereas *Bifidobacterium* members (*p* < 0.05) were reduced in the LC-HIIT group on post-measurement (*p* < 0.05, Table 2). Compared to LC, the LC-MICT group enriched more in *Blautia* members (*p* < 0.05), and both training groups reduced *Alistipes* abundance (*p* < 0.05, Table 2).

**TABLE 2:** Phylum proportion (%) and genus proportion (%) before and after LC with/without training.

	LC (*n* = 11)		LC-HIIT (*n* = 13)		LC-MICT (*n* = 12)		Time	Group	Interaction
	Pre	Post	Pre	Post	Pre	Post	*p*	*p*	*p*
**Phylum proportion (%)**									
Firmicutes	34.1 (23.7)	42.8 (25.7)	38.8 (24.4)	55.2 (28.8)	57.7 (19.7)	68.8 (23.1)	0.691	0.812	0.674
Bacteroidetes	44.4 (30.8)	30.2 (30.8)	43.2 (32.5)	22.4 (28.3)	21.0 (24.1)	8.5 (18.0)	0.665	0.795	0.649
Proteobacteria	8.0 (7.6)	16.1 (17.4)	11.5 (14.1)	17.2 (13.2)	8.0 (5.1)	15.1 (12.8)	0.635	0.818	0.527
Actinobacteria	8.7 (13.5)	5.1 (7.2)	6.2 (8.4)	3.8 (3.3)	13.3 (11.7)	7.6 (8.5)	0.603	0.746	0.594
Fusobacteria	4.8 (9.1)	1.2 (2.2)	0.4 (0.7)	1.4 (4.5)	0.0 (0.0)	0.0 (0.1)	0.674	0.824	0.661
Tenericutes	0.0 (0.0)	0.0 (0.0)	0.0 (0.0)	0.0 (0.0)	0.0 (0.0)	0.0 (0.0)	0.591	0.692	0.145
Other phylum	0.0 (0.0)	4.6 (15.1)	0.0 (0.0)	0.0 (0.0)	0.0 (0.0)	0.0 (0.0)	0.685	0.822	0.682
**Genus proportion (%)**									
Bacteroides	26.0 (24.1)	25.4 (28.8)	27.6 (27.6)	17.7 (24.5)	16.9 (22.4)	7.1 (17.2)	0.109	0.068	0.972
Faecalibacterium	5.9 (5.8)	7.2 (6.2)	7.7 (4.6)	14.3 (19.5)	9.5 (9.5)	11.0 (8.8)	0.753	0.391	0.518
Gemmiger	5.6 (9.3)	5.2 (7.7)	4.6 (5.5)	9.6 (13.9)	7.3 (7.5)	7.3 (5.7)	0.861	0.041	0.823
Clostridium	5.1 (5.4)	5.9 (4.8)	4.9 (6.1)	7.2 (5.6)	5.6 (6.3)	9.0 (4.0)	0.008	0.292	0.486
Prevotella	11.3 (20.1)	1.2 (2.0)	6.8 (14.0)	0.1 (0.1)	0.6 (0.7)	0.2 (0.4)	0.206	0.125	0.484
Ruminococcus	4.0 (4.0)	6.1 (4.9)	5.0 (8.0)	3.7 (3.1)	5.7 (5.8)	7.4 (3.2)	0.147	0.042	0.613
Eubacterium	5.6 (6.3)	4.1 (3.5)	4.1 (3.5)	4.2 (3.7)	4.8 (2.5)	5.4 (2.9)	0.951	0.068	0.759
Roseburia	2.5 (2.4)	1.9 (2.6)	4.3 (4.7)	2.6 (2.9)	6.3 (6.2)	5.8 (5.1)	0.351	0.008	0.944
Bifidobacterium	6.3 (8.2)	2.6 (4.5)	3.8 (4.9)	1.3 (1.7)**	9.0 (7.1)	5.4 (8.0)	0.023	0.048	0.643
Streptococcus	2.5 (2.2)	3.8 (5.4)	4.2 (4.0)	5.3 (7.0)	3.1 (2.6)	4.6 (2.6)	0.248	0.300	0.737
Phascolarctobacterium	1.6 (2.1)	7.0 (10.8)**	1.3 (1.5)	3.1 (3.6)	1.3 (1.4)	5.4 (10.2)	0.023	0.482	0.656
[Ruminococcus]	1.4 (1.3)	2.6 (3.0)	1.4 (0.9)	2.8 (3.3)	2.3 (1.4)	4.5 (4.6)	0.028	0.028	0.968
Lactobacillus	1.2 (1.2)	1.3 (2.1)	4.1 (8.1)	2.3 (5.5)	2.3 (2.6)	1.6 (2.4)	0.439	0.684	0.695
Collinsella	0.8 (1.7)	1.6 (3.5)	1.8 (2.4)	1.7 (2.0)	3.7 (4.5)	4.4 (5.3)	0.713	0.013	0.902
Parabacteroides	1.1 (1.5)	4.3 (7.6)	1.3 (1.5)	3.4 (8.7)	0.9 (1.8)	0.2 (0.1)	0.449	0.023	0.704
Klebsiella	2.6 (7.6)	0.5 (0.7)	2.0 (5.7)	4.0 (6.6)	0.3 (0.6)	1.4 (2.9)	0.741	0.082	0.784
Blautia	1.0 (1.2)	1.0 (0.8)	0.9 (0.6)	1.4 (1.1)	1.5 (0.6)	4.3 (5.6)^∧^	0.194	0.001	0.283
Parasutterella	1.0 (2.3)	1.5 (3.4)	1.6 (2.2)	1.4 (3.2)	3.1 (4.0)	1.9 (3.2)	0.627	0.032	0.805
Dorea	0.7 (1.0)	1.7 (2.5)	1.4 (2.0)	1.7 (2.5)	1.5 (1.2)	1.6 (1.1)	0.680	0.041	0.728
Sutterella	3.0 (7.9)	2.6 (6.2)	0.4 (0.7)	0.1 (0.4)	0.5 (1.2)	0.3 (0.7)	0.706	0.084	0.950
Fusobacterium	3.1 (7.0)	0.8 (1.3)	0.3 (0.7)	0.8 (2.5)	0.0 (0.0)	0.0 (0.0)	0.956	0.118	0.817
[Eubacterium]	0.2 (0.3)	0.5 (1.3)	2.0 (6.7)	0.5 (1.1)	1.9 (6.0)	1.3 (4.1)	0.108	0.578	0.426
Catenibacterium	0.0 (0.0)	0.0 (0.0)	0.8 (2.4)	0.5 (1.5)	2.2 (7.8)	0.9 (3.2)	0.747	0.099	0.943
Alistipes	0.7 (1.0)	2.1 (3.3)	0.4 (1.1)	0.2 (0.7)^∧^	0.8 (1.4)	0.2 (0.2)^∧^	0.416	0.016	0.657
Coprococcus	0.5 (0.9)	0.6 (0.8)	0.5 (0.7)	1.7 (3.8)	1.1 (1.2)	1.2 (1.2)	0.588	0.194	0.914
Megamonas	0.7 (2.4)	1.0 (3.3)	0.7 (1.8)	0.0 (0.0)	0.9 (1.5)	0.1 (0.3)	0.125	0.691	0.699
Lachnoclostridium	0.5 (1.1)	0.4 (0.5)	0.5 (0.6)	0.9 (1.7)	0.5 (0.6)	0.8 (1.1)	0.343	0.667	0.665
Granulicatella	0.6 (0.7)	1.0 (1.2)	0.7 (0.9)	0.7 (0.8)	0.4 (0.4)	0.6 (0.7)	0.524	0.953	0.717
Enterobacter	0.4 (1.1)	0.1 (0.2)	1.2 (3.3)	1.5 (3.3)	0.1 (0.1)	0.2 (0.5)	0.658	0.428	0.960
Other genus	3.5 (2.8)	4.6 (3.6)	2.6 (1.4)	3.6 (2.5)	5.5 (3.8)	3.9 (4.4)	0.915	0.632	0.322

Data are presented as mean (SD). LC, low-carbohydrate diet control group; LC-HIIT, low-carbohydrate diet and high-intensity interval training group; LC-MICT, low-carbohydrate diet and moderate-intensity continuous training group. Compared to pre-measures at **p < 0.01; compared to the LC group at ^∧^p < 0.05.

### Association Between Changes in Gut Microbiota and Cardiometabolic Health

**FIGURE 2:**
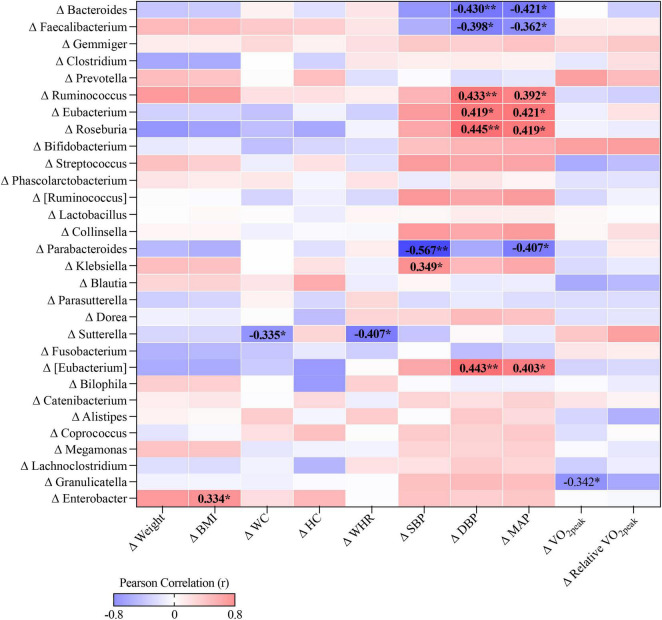
The correlation heat map between changes in cardiometabolic health-related profiles and relative abundance of individual genus, *p < 0.05, **p < 0.01.

In body composition profiles, change of *Sutterella* was negatively correlated with changes of WC (*r* = −0.335, *p* < 0.05) and WHR (*r* = −0.407, *p* < 0.05), while change in *Enterobacter* was positively correlated with change of BMI (*r* = 0.334, *p* < 0.05). Regarding to BP, changes in *Ruminococcus*, *Eubacterium*, *Roseburia*, and unclassified genus from *Eubacterium* were positively associated with changes in DBP (*r* = 0.419–0.445, *p* < 0.05) and MAP (*r* = 0.392–0.421, *p* < 0.05), whereas the changes in *Bacteroides*, *Faecalibacterium*, and *Parabacteroides* were negatively associated with BP changes (*r* = −0.362 to 0.567, *p* < 0.05). Moreover, there was a negative correlation between *Granulicatella* and V̇O_2peak_ (*r* = −0.342, *p* < 0.05, Figure 2).

## Discussion

This study was mainly focused on the effect of LC with/without exercise training on gut microbiota, and the associations between gut microbiota and cardiometabolic health-related profiles. The 4-week LC intervention with additional training or not failed to change the gut microbial diversity or the overall microbial structure in overweight/obese females. Compared to LC intervention only, the LC-MICT group increased the *Blautia* genus, a short-chain fatty acids (SCFAs) producer, while both training groups reduced a type 2 diabetes (T2D) related genus *Alistipes*, which may be beneficial to gut physiology. Moreover, LC- and training-induced changes in BMI, WC, V̇O_2peak_, and BP were associated with a number of specific bacterial genera, suggesting that gut microbiota may play a role in the regulation of cardiometabolic health induced by diet and exercise, though the functional pathways underlying the observed association warrant further study.

Consistent with some previous studies reporting unchanged gut microbial diversity and richness in response to LC in children with epilepsy (13, 17), this study also found that the α-diversity and overall gut microbial structure were unaffected by LC intervention. In contrast, studies in mice showed that LC administration lowered the gut microbial diversity and total bacterial counts (12, 28). In addition to diet, exercise training appears to be another factor contributing to gut microbial changes. People with higher physical activity or fitness levels exhibited a higher diversity in gut microbiome and a greater variety in health-promoting bacteria (29). Short-term training interventions were also reported to increase gut microbial diversity and normalize some of the microbial changes caused by diet-induced obesity in mice (18). However, we failed to find any additional impacts of HIIT or MICT on the gut microbial diversity and overall composition structure in the overweight/obese females. The contradictory findings are likely due to differences in the species (human vs. rodent) of the participants and, more importantly, differences in obesity status, given that the gut microbiota of lean individuals was reported to be more responsive to exercise training than that of overweight or obese individuals (30).

Although without phylum-level alteration, the proportions of several gut microbial genera were changed after intervention. Specifically, the *Phascolarctobacterium* genus was enriched in response to LC and the *Bifidobacteria* genus was reduced after LC-HIIT intervention. The *Phascolarctobacterium* genus is a group of beneficial bacteria capable of producing SCFAs (31), which has been shown to increase with intake of high-fat diets (32). Thus, a sharp increase in dietary fat during LC may partly explain the increased *Phascolarctobacterium* abundance in our dataset. The *Bifidobacteria* genus is generally associated with positive effects on host health and has been frequently used as probiotic components in functional foods (33). However, obese people with higher net fat intake tended to have lower levels of fecal *Bifidobacterium* species compared to lean individuals (34), and this bacteria were reported to decrease with high fat intake (17, 35). Although the *Bifidobacterium* genus was found to be reduced in all groups after intervention in the current study and there was a significant time effect, the *post hoc* test showed that only the LC-HIIT group had significant difference, which may be affected by the small sample size. Moreover, this study used OTU clustering method to determine the taxonomic origin of target gene sequences, which may be not as accurate as amplicon sequence variant (ASV) method (e.g., DADA2) in terms of minimizing the impact of sequencing errors (36), thereby reducing the statistical ability to detect interaction effects.

When exercise training was incorporated, a polysaccharide-metabolizing genus, *Blautia*, was enriched in the LC-MICT group, whereas the *Alistipes* genus was less abundant in both training groups as compared to the LC group. Members of *Blautia* genus are Gram-positive anaerobes that produce lactate and acetate as the major end products of glucose fermentation (37), which was lower in T2D patients (38). Negative associations between the relative abundance of *Blautia* and fasting concentrations of HbA_1c_, glucose (38) and blood lipids (39) were found, suggesting that the *Blautia* genus may be implicated in glucose and lipids metabolism. Consistent with our study, a previous study reported that voluntary exercise increased the *Blautia coccoides*–*Eubacterium rectale* group in mice compared to their sedentary littermates (40). *Alistipes* is a bile-resistant and indole-positive microbiome which can be enriched by high-fat or animal-based diet (39, 41). The relative abundance of *Alistipes* was found to be higher in patients with depression (42) and T2D (43). Therefore, the increased *Blautia* genus and reduced *Alistipes* genus in response to exercise training may be beneficial to glucolipids metabolism. It is noteworthy that the low-volume HIIT (1-min total exercise time) reduced the *Alistipes* genus to the same extent as 30 min of MICT, suggesting that extremely brief training patterns like HIIT are also capable of changing gut microbiota in short term.

We found a positive correlation between an infection-causing bacterial genus, *Enterobacter*, and BMI in the overweight/obese females. In line with our finding, *Enterobacter cloacae* administration was reported to promote subcutaneous fat accumulation and adipocytes hypertrophy, and impair insulin signaling in adipose tissue in mice fed with high-fat diet (44), indicating that *Enterobacter* might affect BMI through promoting adipose tissue hypertrophy and insulin resistance. In addition, negative correlations between *Sutterella* genus and WC as well as WHR were observed. Consistently, a recent study also reported that the *Sutterella* genus was inversely correlated with BMI, WC, body fat mass and body fat percentage in overweight/obese subjects after following a weight-loss eating program with synbiotic supplement (45). The negative association between body composition and genus *Sutterella* seems to indicate a beneficial role of this genus in weight loss, especially in reducing abdominal fat. Studies suggested a critical role of gut microbiota in BP regulation; however, the results were heterogeneous (46–48). SCFAs seem to be involved in microbe–host interactions in BP regulation through interacting with host G-protein-coupled receptors to influence host cells (49). Therefore, SCFA producers, such as *Faecalibacterium*, *Roseburia*, *Ruminococcus*, *Bifidobacterium*, *Akkermansia*, and *Bacteroides* were depleted in hypertension, whereas some *Proteobacteria* and *Bacteroidetes* members, including *Klebsiella*, *Prevotella*, and *Enterobacter* were enriched in hypertension (46). However, the BP-related bacteria genera identified in this study were not completely consistent with those reported in the literature. In the overweight/obese but non-hypertensive cohort, *Ruminococcus*, *Eubacterium*, and *Roseburia* genera were positively correlated with BP, whereas *Bacteroides*, *Faecalibacterium*, and *Parabacteroides* genera were negatively associated BP. The inconsistencies in subjects’ health status (hypertensive in previous studies vs. healthy in our study) and antihypertensive medication use (48) complicated the interpretation of different results among studies. Additionally, the *Granulicatella* genus, identified as a causative agent of endocarditis and bacteremia (50, 51), was found to be negatively correlated with CRF. We have previously found that LC intervention could improve body composition, insulin sensitivity, and BP, and the addition of HIIT or MICT yielded additional benefits in CRF (6–8). This study further revealed that LC- and/or exercise-induced improvements in cardiometabolic health are associated with changes in gut microbiota, suggesting that gut microbiota may serve as potential biomarkers in the regulation of cardiometabolic health induced by LC and exercise. Nonetheless, correlation does not imply causation, and further studies are needed to elucidate the functional pathways underlying the observed correlations and to specify intervention targets for the gut microbiome.

It should be noted that the end products of gut microbiota (fecal metabolite concentrations) were not examined in this study, and the 16S rRNA analysis was unable to identify different microbiome at the species level, thereby further studies interpreting the functional influence of specific microbiome changes on host health are needed. New sequence clustering methods, such as ASV (e.g., DADA2), that attempt to achieve finer taxonomic resolution than traditional OUT, can be used in future study. Moreover, the understanding regarding to the long-term effect of LC on gut microbiota and cardiometabolic health is limited by the short-term nature of this study. Lastly, the small sample size is another study limitation as it was calculated based on the primary outcome of V̇O_2peak_. Further work in a larger cohort and identification of the causal axis of LC–microbiome–health may help to gain more mechanical insights into these preliminary results.

## Conclusion

This study firstly evaluated the effectiveness of LC with/without exercise training on gut microbiota in the obese/overweight females. The 4-week LC intervention with/without additional exercise training failed to change the α-diversity and the overall structure of gut microbiota. After intervention, the LC group increased the *Phascolarctobacterium* genus, and the LC-HIIT group reduced the *Bifidobacterium* genus. Compared to LC intervention alone, groups with exercise training increased a beneficial genus, *Blautia*, and decreased a potential harmful genus, *Alistipes*, which may thus be beneficial to gut physiology. Most importantly, this study sheds new light on several potential microbial targets relating to LC- and/or exercise-induced improvements in BMI, WC, BP, and CRF, though the functional interactions of these association with host health need further study.

## Data Availability Statement

The datasets presented in this study can be found in online repositories. The names of the repository/repositories and accession number(s) can be found in the article/supplementary material.

## Ethics Statement

The studies involving human participants were reviewed and approved by the Panel on Social Science & Humanities Research Ethics of the University of Macau. The patients/participants provided their written informed consent to participate in this study.

## Author Contributions

SS, ZK, and JN: research design and manuscript drafting. ZK: funding acquisition. SS and OL: data collection. SS, QS, and YX: data analysis and interpretation. SS, ZK, OL, QS, YX, and JN: manuscript revision. All authors read and approved the final version of the manuscript.

## Author Disclaimer

The views expressed are those of the authors and not necessarily those of the UM.

## Conflict of Interest

The authors declare that the research was conducted in the absence of any commercial or financial relationships that could be construed as a potential conflict of interest.

## Publisher’s Note

All claims expressed in this article are solely those of the authors and do not necessarily represent those of their affiliated organizations, or those of the publisher, the editors and the reviewers. Any product that may be evaluated in this article, or claim that may be made by its manufacturer, is not guaranteed or endorsed by the publisher.
